# Population-Based Disparities in U.S. Urban Heat Exposure from 2003 to 2018

**DOI:** 10.3390/ijerph191912314

**Published:** 2022-09-28

**Authors:** Daniel P. Johnson

**Affiliations:** Department of Geography, Indiana University—Purdue University at Indianapolis, Indianapolis, IN 46202, USA; dpjohnso@iu.edu

**Keywords:** surface urban heat island, racial disparity, thermal exposure, environmental justice, social justice, climate justice, Bayesian spatial temporal modelling

## Abstract

Previous studies have shown, in the United States (U.S.), that communities of color are exposed to significantly higher temperatures in urban environments than complementary White populations. Studies highlighting this disparity have generally been cross-sectional and are therefore “snapshots” in time. Using surface urban heat island (SUHI) intensity data, U.S. Census 2020 population counts, and a measure of residential segregation, this study performs a comparative analysis between census tracts identified as prevalent for White, Black, Hispanic and Asian populations and their thermal exposure from 2003 to 2018. The analysis concentrates on the top 200 most populous U.S. cities. SUHI intensity is shown to be increasing on average through time for the examined tracts. However, based on raw observations the increase is only statistically significant for White and Black prevalent census tracts. There is a 1.25 K to ~2.00 K higher degree of thermal exposure on average for communities of color relative to White prevalent areas. When examined on an inter-city basis, White and Black prevalent tracts had the largest disparity, as measured by SUHI intensity, in New Orleans, LA, by <6.00 K. Hispanic (>7.00 K) and Asian (<6.75 K) prevalent tracts were greatest in intensity in San Jose, CA. To further explore temporal patterns, two models were developed using a Bayesian hierarchical spatial temporal framework. One models the effect of varying the percentages of each population group relative to SUHI intensity within all examined tracts. Increases in percentages of Black, Hispanic, and Asian populations contributed to statistically significant increases in SUHI intensity. White increases in population percentage witnessed a lowering of SUHI intensity. Throughout all modeled tracts, there is a statistically significant 0.01 K per year average increase in SUHI intensity. A second model tests the effect of residential segregation on thermal inequity across all examined cities. Residential segregation, indeed, has a statistically significant positive association with SUHI intensity based on this portion of the analysis. Similarly, there is a statistically significant 0.01 K increase in average SUHI intensity per year for all cities. Results from this study can be used to guide and prioritize intervention strategies and further urgency related to social, climatic, and environmental justice concerns.

## 1. Introduction

Significant disparities in community-level heat exposure have been identified in many urban locations in the United States [1,2,3,4,5]. Due to variations in the surface urban heat island (SUHI) effect, communities that have historically been marginalized are exposed to significantly higher SUHI temperatures than predominantly White communities within the same city. However, we lack a clear understanding of the temporal and spatial trends in heat exposure within these communities and cities. For example, are SUHI temperatures in predominantly Black communities increasing or decreasing through time? A strong contributor to these disparities is the effects of residential segregation and its influence on disparate thermal exposure. These effects need further quantification, especially longitudinally. Uncovering trends in inequitable exposure and validating the inequities through time and space will further efforts to combat environmental, social, and climatic injustice. Armed with these types of analyses policy-makers and community planners can focus on the most inequitably exposed members of urban populations.

This study examines the spatial-temporal aspects of urban heat exposure within the top 200 most populous cities in the US. The analysis is performed at the census tract-level across all cities and for each city as a whole. Utilizing measurements of the SUHI from 2003 to 2018 and applying a Bayesian hierarchical spatial temporal modelling approach, enables the examination of SUHI intensity measurements relative to US Decadal Census data, quantifying the predominant racial composition of census tracts and the level of segregation in the respective city. Tracts are stratified into the prevalence of the population-based groups of White, Black, Hispanic, and Asian. This methodology allows for the determination of the temporal trends in heat exposure in affected areas based on demographic prevalence. Moreover, it will foster a more complete understanding of the disparity in thermal exposure through time and hopefully, catalyze efforts to improve local environments in affected areas and promote social, climatic and environmental justice. This study should add further weight and urgency to similarly inclined environmental justice pursuits especially related to heat health risk and the inequitable distribution of environmental health hazards.

### 1.1. Disparities in Urban Heat Exposure in the U.S.

Numerous studies have shown there is a significant disparity in heat exposure within U.S. cities between White and historically marginalized communities [1,2,3,4,5]. Harlan et al., observed in 2007 that affluent predominantly White communities were more likely to live in vegetated and less climatically stressed neighborhoods than those in poor Latino communities within Phoenix, AZ, U.S. [1]. In a study focusing on Richmond, VA, U.S., Saverino et al., found census block level areas occupied by people of lower SES and/or those historically marginalized disproportionately experienced higher temperatures and higher impacts on health [5]. Mitchell and Chakraborty observed that census tracts with higher percentages of people of color and higher percentages of those in poverty had significantly higher temperatures than predominantly White census tracts in Pinellas County, FL, U.S. [3]. In a separate study focusing on the three largest U.S. cities (New York City, Los Angeles, and Chicago) they also found significant bivariate statistical associations between lower SES, historical marginalization and urban heat risk [4].

Expanding on their 2015 study and including 20 of the largest US cities along with a measure of residential segregation, Mitchell and Chakraborty, found variables representative of lower SES to be consistently statistically associated with higher heat exposure. However, belonging to a historically marginalized group and residential dissimilarity (a measure of segregation) has a varied but significant relationship using a multilevel modeling approach [6]. In a national-level study using block group-level data across 304 metropolitan areas in the U.S., Jesdale, Morello-Frosh, and Cushing found that non-Hispanic Black populations were 52%, non-Hispanic Asians 32% and Hispanics 21% more likely to live in areas with higher concentrations of “heat risk related land cover” [7]. These conditions increased with increasing degrees of residential segregation. This was determined using the 2001 National Land Cover Dataset to identify ‘heat risk related land cover” and 2000 US Decadal Census data. Hsu et al., utilizing the same SUHI dataset used in the present study and 2017 ACS 5-year data, found that on average persons of color live in census tracts with higher SUHI intensity than non-Hispanic Whites in all but 6 of the 175 largest urbanized areas in the continental US [2].

One of the primary concerns with the disparity in urban heat exposure is the increased risk of heat-related impacts on human health. Heat is a leading weather-related cause of mortality and morbidity [8,9]. Heat-related illness is a spectrum of conditions that are brought about by exposure to extreme temperatures, including heat edema, dehydration, heat syncope, and life-threatening heat stroke [10]. Heat is also known to exacerbate or contribute to a number of chronic and acute health conditions that can lead to amplified illness or death. Cardiovascular disease, Chronic Obstructive Pulmonary Disease (COPD), asthma, chronic kidney disease, rheumatoid arthritis, type 2 diabetes, migraines, and mental illness are only a sample of the more prevalent conditions exacerbated by extreme heat [8,11,12,13,14,15,16,17,18,19]. These and other health impacts from thermal exposure, especially during extreme heat events, can quickly overload emergency medical management centers [20]. Further complicating matters, populations that are frequently exposed to extremes in temperature, especially in urban areas, are more prone to the effects of extreme heat due to sociodemographic characteristics known to increase vulnerability [21,22,23,24]. Therefore, further understanding and lessening the disparity in urban thermal exposure should tend to lower the risks of heat-related morbidity and mortality.

### 1.2. Study Motivations

The principal intent of this study is to add to the understanding of the dynamic nature of inequitable urban thermal exposure. As of summer 2022, there were no readily available studies investigating this issue through time. The aforementioned cross-sectional studies are very important and shed light on the significant disparity in urban temperature exposure in the U.S. However, a major limitation is they are “snapshots” in time. Longitudinal examinations allow for the investigation of trends in the data and offer further validity of observations through a number of temporal periods. Such an investigation can offer clues to whether or not these thermal exposure inequities are increasing or decreasing through time. Furthermore, longitudinal investigations that contain an element of space (or geography) require complex methodologies that can account for spatial and temporal autocorrelation within the data, considerations of which methods such as ordinary least squares regression cannot account for. Methodological techniques must account for spatial, temporal and spatial–temporal interactions. There are often high degrees of spatial structure or spatial autocorrelation (a grouping or clustering of similar values) within these types of data. Similarly, the temporal structure can exist from one time period to the next. There may even be structures in neighboring areas during a previous temporal period that interact through space and time. Such interactions, when not accounted for, can lead to a number of issues (it is also often interesting to explore these interconnections). Potential issues include the inflation of variance that can impact coefficients, leading to erroneous calculations of statistical significance. Bayesian hierarchical spatial temporal methods can account for these considerations in a robust and elegant manner. These techniques are often employed in disease mapping studies and the procedure outlined in the methods section is primed, with some procedural modifications, for the inclusion of heat health data and should allow future investigators to modify the technique with the inclusion of such data.

## 2. Methods

### 2.1. Data Collection and Descriptive Comparison

Demographic and SUHI intensity data were collected from the top 200 most populous cities in the US defined by the US Decadal 2020 Census [8,9,25,26]. There is some overlap between variables since “Black” includes Hispanic and non-Hispanic Black persons. “Hispanic” also includes Black Hispanics. At the time of collection, data were not available on Black Hispanics so it was not possible to account for them specifically. The counts for the total population and populations of White, Black, Hispanic, and Asian persons were retrieved for the respective census tracts of each city. These counts were also used to create a percentage of each population group for each census tract. There were a total of 44,622 census tracts selected at this stage.

Land surface temperature data, utilizing summertime SUHI intensities were collected from the “Yale Center for Earth Observation (YCEO) Surface Urban Heat Islands: Pixel-Level Composites of Yearly Summertime Daytime and Nighttime Intensity” dataset on Google Earth Engine™ [27,28]. These data are pixel level, 300 m spatial resolution, composites of average daytime and nighttime SUHI temperature differential (intensity) within the urban areas during the summertime. SUHI intensity is calculated by comparing the pixels in the urban spaces to those that are in the rural adjacent or background landscape creating a SUHI intensity metric. SUHI intensity of an individual pixel is a function of the representative urban pixel’s land surface temperature (LST) relative to the LST of the neighboring rural reference or non-urbanized neighboring pixels, (LST_Rural_) the mean LST of the neighboring non-urbanized area [29,30].

$$\mathrm{SUHI}\;\mathrm{Intensity}\;={\mathrm{LST}}_{\mathrm{Urban}\;\mathrm{Pixel}}-{\;\mathrm{LST}}_{\mathrm{Rural}}$$

The delineation between what is urban and non-urban or rural is determined by a simplified urban extent method using urban extent data from Natural Earth™, which is a combination of urban land databases from Schneider et al. and Oak Ridge National Laboratory’s LandScan product [31,32,33]. Thermal measurements are derived from MODIS 8-day land surface temperature products, terrain elevation data, and land cover derived from the European Space Agency [29,30,34,35,36]. Only daytime measurements during the summer are included in this study.

**Figure 1:**
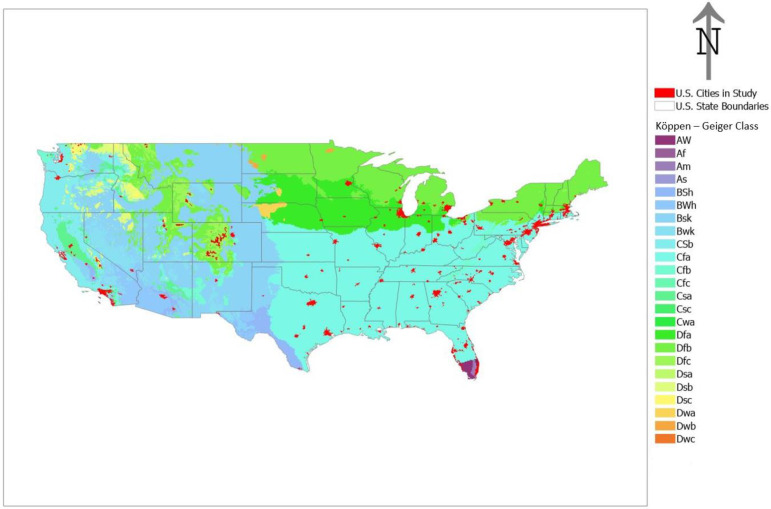
Urban areas in the study (n = 191) and their respective Köppen–Geiger climate classification. All cities and census tracts were assigned to their respective climate zone to account for climatic variations in the data.

**Figure 2:**
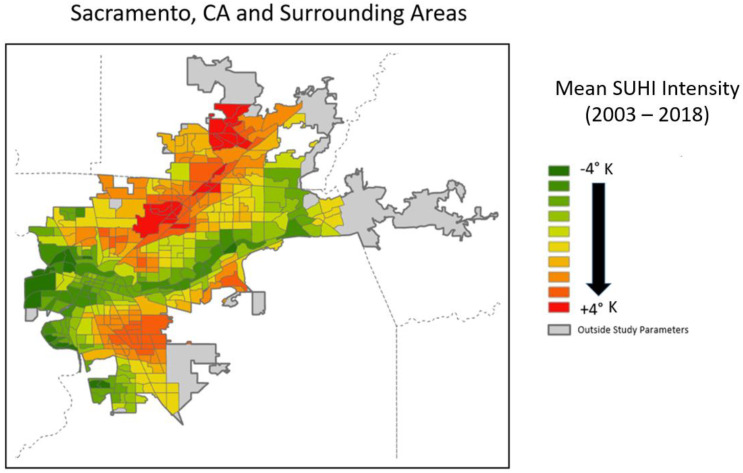
Example city used in the study demonstrating the overlay, or lack thereof, between the SUHI intensity dataset (classed) and census designated urban areas. Only census tracts that are spatially aligned with the SUHI intensity dataset are used in the study. Dashed lines represent county boundaries, also notice the presence of spatial autocorrelation or the clustering of similar values.

The SUHI intensity dataset only contained data for 191 of the 200 selected cities (Figure 1). Furthermore, of those 191 identified, not all census tracts were congruent with the SUHI boundary for each city; some of the tracts lie outside the boundary and no temperature data were available for them (cf: Figure 2). If any pixel from the SUHI intensity dataset was within a census tract the tract was selected for the study. Of the 44,622 census tracts initially extracted, 44,476 tracts remained after accounting for this lack of overlap. These tracts include 48.11% of the total US population: 70.05% of the Black, 66.31% of the Hispanic, 78.80% of the Asian and 44.03% of the White population (as of 2020).

A zonal mean function was used to calculate the yearly mean summertime SUHI intensity for each census tract (see Figure 2). In order to incorporate another layer of analysis and to compare percentiles of thermal exposure, census tracts were categorized by their percentile ranking of SUHI intensity. This allows for the examination of the percentages from each population group living in the different thermal percentiles. For example, the percentage of Hispanic persons living in the 95th percentile of SUHI intensity.

Prevalence was the primary method used to categorize the demographic makeup of each census tract. For example, if there were more Hispanic people in a census tract than any other population group, it was categorized as a “Hispanic Prevalent” tract. There are a total of 27,338 White, 6597 Black, 7917 Hispanic, and 2624 Asian prevalent tracts in the study. Categorization in this way enables comparisons between population groups and the degree of thermal exposure. Furthermore, prevalence is an easy-to-calculate measurement that will capture the group with the highest population within the tract and it is easy to reproduce. However, it does have limitations for categorization. Some categories could contain higher percentages of the secondary population group than are within their primary categorization. For example, a tract that has 51% Hispanic and 49% White populations would be considered a Hispanic prevalent tract. Another tract may have 26% White, 24% Black, 25% Hispanic and 25% Asian populations and would be considered a White prevalent tract. The latter example may not necessarily have a higher population of White persons than the prior categorized tract. These limitations should be kept in mind when interpreting the results.

A separate inter-city dataset was created where all 44,476 census tracts were aggregated by city. This allows for the determination of which cities have the highest levels of SUHI intensity exposure for each demographic group. The mean SUHI intensity for each city was calculated by averaging the SUHI intensity for all representative tracts for each year. The rankings for the cities based on each demographic group are presented in dot plot diagrams. A segregation index using the multi-group version of the Duncan Dissimilarity (*D*) metric was also calculated at this step using the counts of each population group [37,38]. This index relates the amount of each group’s population that would have to move between tracts for each census tract to have the same percentages of population distribution as the respective city. Values range between zero and one, zero being perfect integration and one being absolute segregation. All 191 cities were included in these calculations. The index was calculated using the R statistical platform and the OasisR package [39].

### 2.2. Modeling

Two models were developed using a Bayesian spatial temporal ecological regression methodology. One model applied SUHI intensity across all census tracts from 2003 to 2018 (44,476 in number for 16 years) as the response with the percentages of each population group as explanatory variables. The second model utilized the inter-city dataset (n = 191 × 16 years), with mean city-wide SUHI intensity as the response and the *D* segregation index as an explanatory variable.

For the first model, SUHI intensity *T* at location *i*, *T_i_*_,_ was specified with a Gaussian distribution.

$$T_{i}\;\sim \;Normal\left(\theta_{i},\sigma_{T}^{2}\right),\;i=1,\ldots ,44,476$$

$$\theta_{i}\;\sim \;Normal(\mu_{i},\sigma_{U}^{2}),\;i=1,\ldots ,44,476$$

Including covariates—percentages of the four population groups—in a vector, *d_i_*, containing the intercept and *β* as a coefficient vector, the overall spatial model with fixed effects is:

$$\theta_{i}=d_{i}\beta +S_{i}+U_{i}$$

*S_i_* and *U_i_* are the spatially structured and unstructured effects utilized in the Besag, York, Mollié (BYM) model [40]. In this study the parameterization of the BYM model proposed by Simpson at al. which assigns penalized complexity (PC) priors is used [41]. Typically referred to as the “BYM2” model, it utilizes scaled spatially structured and unstructured components ($S_{*}\;\mathrm{and}\;U_{*}$) and is defined as:

$$\theta_{i}=d_{i}\beta +\frac{1}{\sqrt{\tau_{b}}}\left(\sqrt{1-\varphi }\;S_{*}+\sqrt{\varphi }U_{*}\right)$$

$\tau_{b}$ is a precision parameter which controls the contribution of marginal variance to the structured and unstructured components. The mixing parameter φ controls the portion of variance within the structured random effect. Thus when φ = 1 the effect is equal to the spatially structured component and when φ=0 it is equivalent to unstructured spatial noise. This scaled specification has been shown to be superior to the often-employed unscaled BYM model and as its name suggests penalizes complexity [41,42]. Additionally, it more easily enables interpretation of the structured and unstructured effects of the model. These random effects account for variables not in the model that exhibits spatial structure and unstructured spatial noise.

The above spatial specification for *θ_i_* is extended into the spatial-temporal domain by the addition of further random effects.

$$\theta_{it}=d_{i}\beta +\frac{1}{\sqrt{\tau_{b}}}\left(\sqrt{1-\varphi }\;S_{*}+\sqrt{\varphi }U_{*}\right)+\gamma_{t}+\omega_{t}+\delta_{it}$$

$\gamma_{t}\;\&\;\omega_{t}$, represent the temporally structured and temporally unstructured random effects respectively. These account for variables and effects that would exhibit temporal structure and noise. Typically, $\gamma_{t}$, is modeled as a conditional autoregressive random walk of either order one or two (RW1, RW2), but there can be additional specifications (i.e., seasonal, cyclical). In this study, the temporally structured effect is modeled as RW1 with cyclic adjacency (to account for any cyclic tendencies of the data), with $\omega_{t}$ as a Gaussian exchangeable IID ($\omega_{t}\;\sim \;Normal\;(0,\frac{1}{\tau_{\omega }}))$. The spatial temporal interaction component $\delta_{it}$, represents a vector which varies through space and time. This allows for deviations from the spatial and temporal structure and conveys both active spatial changes from one time frame to another and temporal patterns from one area to another [41]. The spatial temporal interaction utilized was Knorr–Held Type 1, thereby allowing no restraints on spatial variations locally and globally through time [43]. Variations in Köppen–Geiger climate zones and population density were accounted for in the models as fixed effects (IID). The percentages of each population group (percentage (%) White, % Black, % Hispanic, And % Asian) were included as explanatory (independent variables). These were standardized to aid in interpretation and due to the presence of interaction terms in the models. Model calculations were performed in the Indiana University High Performance Computing environment using the R statistical platform and the R—INLA package [44,45].

The second model utilized the inter-city dataset and fit daytime SUHI intensities averages across each city as the response with *D* (n = 3056; 191 cities × 16 years) as a fixed effect. SUHI intensity was specified as a Gaussian (normal) distribution:

$$T_{i}\;\sim \;Normal\left(\theta_{i},\sigma_{T}^{2}\right),\;i=1,\ldots ,3065$$

$$\theta_{i}\;\sim \;Normal\left(\mu_{i},\sigma_{U}^{2}\right),\;i=1,\ldots ,3056$$

*D* was assigned a $\beta_{D}\;\sim \;Normal\left(\mu ,\sigma \right)$ prior distribution. The random effect specifications for the first model, BYM2, $\gamma_{t},\;\omega_{t},\;\delta_{it}$, were also utilized here only on an inter-city basis. This model similarly accounted for population density and variations in Köppen–Geiger climate zones between cities.

## 3. Results

### 3.1. Census Tract and City-Level Comparisons

Table 1 presents the descriptive statistics of mean yearly summertime SUHI intensity and demographic makeup for all census tracts in the study from 2003 to 2018. The maximum SUHI intensity observed in any tract was 11.06 K, with a −13.63 K minimum. The mean SUHI intensity of all included tracts was 2.05 K. The mean percentage of White persons living in all tracts was 51.16%, with 17.87% Black, 21.99% Hispanic, and 8.10% Asian populations.

Descriptive statistics for mean yearly summertime SUHI intensity for the census tracts after grouping by prevalence are shown in Table 2. The mean SUHI intensity for White prevalent tracts is 1.46 K with Black and Hispanic prevalent tracts statistically significantly higher at 3.08 K and 3.15 K respectively. SUHI intensity in Asian prevalent tracts (2.73 K) was also higher than White prevalent tracts but is not statistically significant based on the Kolmogorov–Smirnov test. The maximums for all groups were within 1 K of each other but White has a lower minimum value than the alternative prevalent tracts.

**Figure 3:**
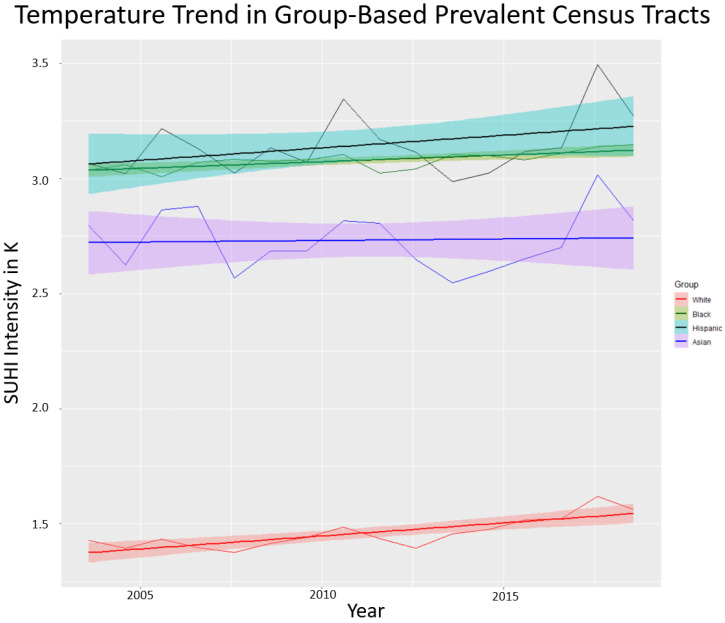
Daytime SUHI intensity for prevalent tracts of each demographic group through time. These are the observed raw (non-modeled) values averaged yearly for census tracts classified by prevalence of each demographic group from 2003 to 2018. The line of best fit and confidence intervals are determined by univariate ordinary least squares linear regression.

Figure 3 presents mean yearly summertime SUHI intensity data through time for the prevalent census tracts of each population group. All four groups show a slight trend of increasing average SUHI intensity through time. Univariate linear regression was used to determine a line of best fit for each time series: White prevalent tracts $\left(y=1.36+0.011x\right)$, Black prevalent tracts $\left(y=3.03+0.006x\right)$, Hispanic prevalent tracts $\left(y=3.05+0.011x\right)$, and Asian prevalent tracts $\left(y=2.72+0.001x\right)$, and the associated 95% confidence intervals (shown in shading). These increases are only statistically significant for the White and Black prevalent tracts. Most notable is the difference in SUHI intensity exposure between each grouping. Asian, Hispanic, and Black prevalent tracts are exposed to significantly higher SUHI intensities than White prevalent tracts, which are on average more than 1.36 K–1.69 K cooler than their counterparts are from year to year.

The percentiles of mean yearly summertime SUHI intensity exposure for each population group is shown in Table 3. Table 3 is organized by the percentage of each group’s population that resides in census tracts categorized by the varying percentiles of SUHI intensity. Consistently, lower percentages of White population reside in hotter tracts. The three alternative groupings show predominantly similar percentages of persons living in the percentile categorizations.

### 3.2. City-Level Comparisons

City-level descriptive statistics are shown in Table 4. These were tabulated after grouping all census tracts by their respective city/municipality into 191 unique observations for each of the 16 years where SUHI intensity data are available. The mean yearly summertime SUHI intensity for all cities was 1.50 K with a maximum of 7.31 K and minimum of −2.61 K. *D* varied among cities with a mean of 0.38, a maximum of 0.63 and minimum of 0.08.

**Figure 4:**
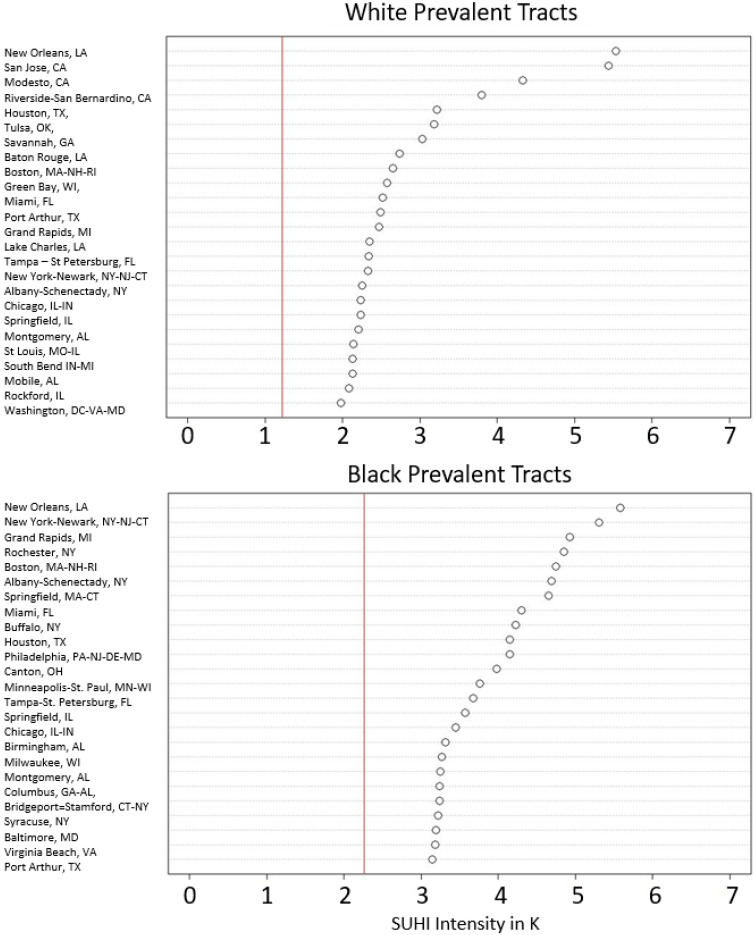
Top 25 cities ranked by highest SUHI intensity for census tracts classified as White and Black prevalent. Circle is the average SUHI intensity for the prevalent tracts of the respective city. Red vertical line represents the overall average SUHI intensity for the prevalent tracts of the demographic group. (Note: Black, Asian, and Hispanic prevalent tracts did not exist in all cities, however, each city did contain White prevalent tracts).

**Figure 5:**
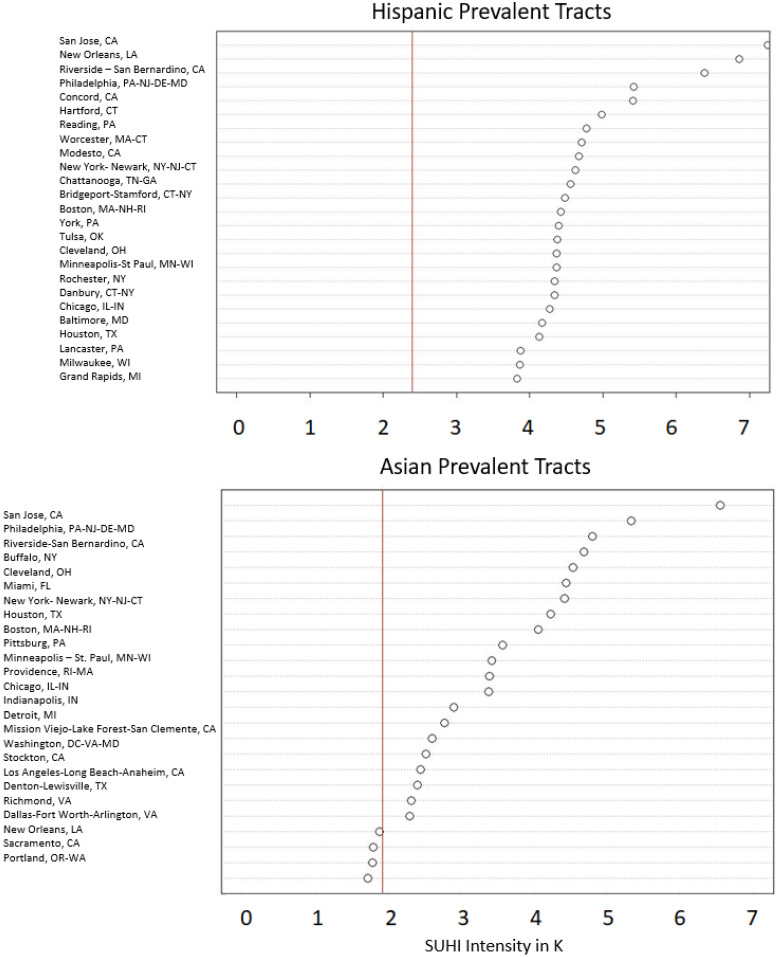
Top 25 cities ranked by highest SUHI intensity for census tracts classified as Hispanic and Asian prevalent. Circle is the average SUHI intensity for the prevalent tracts of the respective city. Red vertical line represents the overall SUHI intensity average for the prevalent tracts of the demographic group.

Mean yearly summertime SUHI intensity was averaged over the 16-year time frame for prevalent census tracts in each city. This was used to examine which cities had the highest levels of SUHI intensity exposure for each prevalent demographic grouping. Comparisons of the 25 most intense cities are shown in Figure 4 and Figure 5. The corresponding dot plots are organized by each prevalent demographic group and plot the mean SUHI intensity for their prevalent tracts in each city. The vertical red line in each plot represents the average SUHI intensity of the census tracts for the respective population group through all 16 years. Averages are significantly higher for Black (K-S *ρ* = 0.0001) and Hispanic (K-S *ρ* = 0.0001) groupings compared to White; the Asian percentage was not statistically different from the White (K-S *ρ* = 0.43), likely due to the lower number of observed cities with Asian prevalent tracts. New Orleans, LA, has the highest SUHI intensity for Black prevalent tracts at nearly 6 K. San Jose, CA, is highest for Hispanic (>7 K) and Asian prevalent tracts (<7 K). New Orleans also has the greatest SUHI intensity for White prevalent tracts with an approximate difference of ~5.5 K. As a further example and using 4 K as an arbitrary SUHI intensity threshold, three cities exceed this intensity on average for White prevalent tracts, 12 cities for Black prevalent tracts, 22 for Hispanic prevalent tracts, and nine for Asian prevalent tracts.

### 3.3. Ecological Regression Models

**Figure 6:**
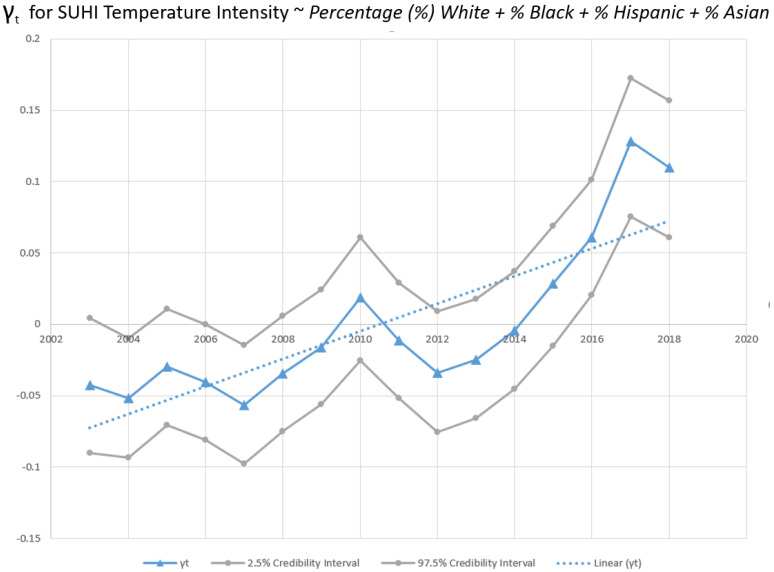
Temporal structure of Bayesian hierarchical spatial temporal model for census tract-level SUHI intensity with percentage of each demographic group within the tract as an explanatory variable. γt is the temporally structured effect modeled as a random walk of order 1 (RW1). Line of best fit is determined by ordinary least squares univariate linear regression. n = 711,616. Gray lines are 95% credibility values. Dased line is the line of best fit derived from ordinary least squares linear regression.

Results of the ecological regression models are shown in Table 5. The first model utilizes mean yearly summertime SUHI intensity by census tract as the response and the percentages of each population group as an explanatory variable. All four population groups had a statistically significant association with average daytime SUHI intensity. Of the independent variables, the Black population percentage had the highest posterior estimated β of 0.42. The Hispanic and Asian population percentages had a mean β of 0.23 and 0.14 respectively. The White population had a negative association with average daytime SUHI intensity with a mean posterior β of −0.25. The temporally structured effect, γ_t_, modeled as a random walk order one, exhibited a statistically significant increase in SUHI intensity through a time of approximately 0.01 K per year on average (Figure 6).

**Figure 7:**
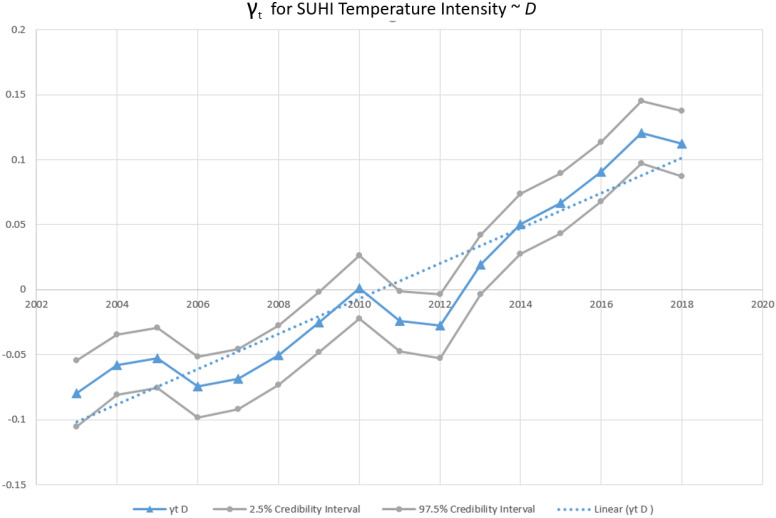
Temporal structure of Bayesian hierarchical spatial temporal model for city-level SUHI intensity and *D* Multi-Group Dissimilarity Index as an explanatory variable. γt is the temporally structured effect modeled as a random walk of order 1 (RW1). Line of best fit is determined by ordinary least squares univariate linear regression. n = 3056. Gray lines are 95% credibility values. Dased line is the line of best fit derived from ordinary least squares linear regression.

The second model used the *D* index as an explanatory variable with SUHI intensity averaged across each city throughout the study time-period (n = 3056) as the response. The coefficient, confidence envelope and intercept are shown in the lower section of Table 5. The second model presents a statistically significant association between *D* and mean yearly summertime SUHI intensity with a mean posterior β of 0.42. γ_t_, the temporally structured effect, displayed a similar pattern to the census tract-level analysis with a statistically significant approximated increase of 0.01 K per year on average (Figure 7).

## 4. Discussion

### 4.1. Census Tract-Level Comparisons

Examined temporally, SUHI intensity increases through a time when the measurement is averaged for all tracts and categorized by prevalent population group (See Figure 3). However, the increase is only statistically significant for the White and Black prevalent census tracts. There is a high degree of variability from year to year among the Hispanic and Asian prevalent tracts, which is responsible for the lack of statistical significance. Reasons for these variations in SUHI intensity are hard to isolate but may be due to the overall residential mobility of these populations in urban areas relative to Black and White prevalent groupings [46,47]. Research has shown that residential mobility (household-level moving) patterns among Hispanic (and Black) urban residents work to reinforce segregation and this may be a factor in the variations observed [48]. Home and/or landscape improvement with varying owners may affect the local thermal environment. Additionally, there may be other spatial intricacies within the census tracts of these groups that contain more variability in land cover types, thereby adding to the higher variance. This would likely be especially variable in many locations in the Western U.S that lack some of the forest cover present elsewhere.

The results of the census tract-level analysis also add further support to previous work demonstrating disparities in temperature exposure between White communities and those of color [1,2,3,4,5,7]. On average across all census tracts examined, there is a greater than 1.25 K–2.0 K difference between them (cf: Table 2). These observations add further support to Hsu et al. demonstrating that historically marginalized groups are exposed to higher SUHI intensities, in the present case approaching >2 K in some cases on average [2]. However, Hsu finds that Black communities on average have the highest degree of inequitable thermal exposure followed by Hispanic ones. The findings presented before fitting the models, which are the raw observations of average temperature across all prevalent tracts, where population density, Köppen–Geiger climate zone, temporal and spatial effects are not yet accounted for, show Hispanics on average to have the highest thermal exposure followed by Black and Asian, with White community exposure negligible by comparison. Furthermore, Hsu et al. use a weighted average of the SUHI intensity for each tract based on the number of individuals in each demographic group. The methodological differences between using a weighted average and prevalence could explain some of the differing observations.

To illustrate the significance and scale of difference in exposure to SUHI intensity between the demographic groupings and utilizing the trend (slope of the interpreted line of best fit) in intensity exposed to each demographic group temporally, it is possible to calculate the time it would take White prevalent tracts to reach temperature parity with their historically marginalized counterparts. Based on this analysis and given the temperature average in 2018 and assuming the trend remains stable through time, it would take until the year 2363 for White prevalent tracts to reach thermal parity with Black prevalent tracts and 2154 for Asian prevalent tracts. White prevalent tracts would never reach thermal parity with Hispanic tracts since they are on average warming the same amount 0.011 K per year. It should again be strongly emphasized, these dates are useful only for comparative/illustrative purposes but they do underscore the inequitable distribution in thermal exposure through time within different communities. Perhaps such an analysis would be useful for policymakers to exemplify the significant thermal disparity between groups.

Examining the breakdown of the percentages of the four demographic groups residing in the varying percentile rankings of thermal exposure, significantly more historically marginalized residents, by percentage, live in hotter census tracts, again supporting many of the previous studies. In the 90th percentile of SUHI intensity, 12.91%, 14.81%, and 12.72% of Black, Hispanic, and Asian study participants, respectively, reside. By comparison, only 5.43% of the White population in the study inhabit areas equal to or in excess of the 90th percentile in SUHI intensity. These numbers fundamentally triple when examining the 75th percentile. The coolest, 5th, percentile examined, includes 6.32% of White persons in the study compared to 2.09% Black, 3.1% Hispanic, and 7.17% Asian persons. The fact that the Asian percentage is higher in this percentile conceivably illustrates some of the complexities of Asian socioeconomic statuses in the U.S. [49]. If the Asian variable was more stratified, (i.e., split into countries or regions of origin such as East Asia or South Asia, as is sure to come in future census data releases) examining this outcome in more depth would be possible.

### 4.2. City-Level Comparisons

The city-level analysis continues to further validate prior studies [1,2,3,4,5,7]. The SUHI intensity for White prevalent tracts averaged and grouped by the city is over 1 K cooler than Black and Hispanic groupings, which is observable through the dot plots in Figure 4. New Orleans, LA, has the highest mean yearly summertime SUHI intensity for both White and Black prevalent tracts. New Orleans is the second-ranked city for Hispanic communities, which are nearly 1 K warmer than their White or Black prevalent counterparts. However, as the city-averaged temperature for White prevalent groupings rapidly decreases, Black prevalent groupings do not. Seven cities are listed as more than 3 K hotter than their background temperature for associated White communities but there are more than 30 cities for Black, 50 for Hispanic, and 16 for Asian communities surpassing this 3 K threshold.

San Jose, CA, has the highest SUHI intensity for both Hispanic and Asian communities and the third for the White community. Based on the 2020 census tract-level data, San Jose, has an Asian plurality population of approximately 38% and is also the location with the largest SUHI intensity for Asian-Americans. Many of these residents live in “Japantown” or “J town” an area with little vegetation and a high density of impervious surfaces which contribute to the thermal environment [50].

Notable among the Hispanic rankings is there are only five cities west of the Mississippi River: four in California, one in Texas in the top 25. Most of the remaining cities are within the Midwest and east coast of the US. Based on this observation, Hispanic populations east of the Mississippi River are exposed to higher SUHI intensities than in the Western U.S., at least within the top 25 cities. Notably missing in the rankings for Hispanic exposure is Phoenix, AZ, which has a high degree of heat-related issues every year and a large Hispanic population. The likely reason for the absence of Phoenix among this ranking is the listing is not based on absolute SUHI temperature but on SUHI temperature differential (intensity) relative to the surrounding environment. The UHI in Phoenix does not show the same degree of intensity during the daytime, compared to the background temperature, as heat islands in the Midwest or US east coast and south due to the paucity of significant surrounding vegetation [51]. The more arid, less vegetated surrounding land cover contains components that also absorb energy, thereby creating a less drastic temperature differential relative to urban heat islands in the eastern U.S. [52,53]. This could also help explain the number of cities east of the Mississippi River in the overall rankings since these are likely to have more significant amounts of vegetation in the background environment leading to higher SUHI intensity measurements.

### 4.3. Models

Both ecological regression models created in this study provided statistically significant results and quantified variations in SUHI exposure for the examined demographic groups. The first model, revealing the relationship between SUHI intensity and the percentage of each population group by census tract, sets SUHI intensity in areas of a city with no population (zero for all groups) equal to 2.05 K hotter on average than the background temperature, based on the intercept of the model. Therefore, areas located within the city are on average are 2.05 K warmer than their background environment and each demographic group’s prevalent tracts (and the populations within them) are exposed to differing levels of SUHI intensities. For each one standard deviation increase in the White population (26.69%) for a census tract, SUHI intensity tends to decrease by 0.25 K on average. Standard deviation increases in the Black population (23.44%) witness SUHI intensity increases of 0.42 K on average. Hispanic increases in percentage by 23.02% saw an SUHI intensity increase of 0.23 K and on average, an 11.50% increase in the Asian population saw an increase in SUHI intensity by 0.14 K.

These models do not imply causation or suggest that increases in one population group would change the SUHI intensity. For example, the gentrification of a neighborhood with an influx of affluent White populations would not necessarily lower the SUHI intensity of the affected area unless there was an emphasis placed on greening [54]. This discussion is simply demonstrative of the trends and differences in thermal exposure, as measured by SUHI intensity, of the different demographic groups. Variations in Black population percentage have the strongest effect on the model suggesting a higher degree of inequitable thermal exposure among the urban Black population than all demographic groups examined. These results unequivocally support Hsu et al. [2] and suggest their findings hold through time. Specifically that Black residents experience the highest average SUHI exposure, Hispanics the second highest and non-Hispanic Whites with the lowest degree of exposure [2]. The present observation for Asian populations also supports the findings of Jesdale et al., where Asian populations were more likely than the White population to live in more stressful thermal environments but less likely than the Black population [7]. However, Jesdale et al. [7] found a lower probability for Hispanics to be in stressed thermal environments. Findings here contradict this observation and find Hispanics to be highly likely to reside in thermally extreme environments based on the prevalence categorization.

SUHI intensity is increasing throughout the study period as evidenced by the temporally structured effect in the model (Figure 4) and is statistically significant as measured by the 95% credibility envelope. On average, 2003–2018 witnessed a 0.01 K per year increase in SUHI intensity within the census tracts examined. This aligns with the earlier finding where raw SUHI intensity measurements were shown to be increasing (Figure 3). The earlier finding showed a statistically significant increase for only Black and White prevalent tracts, but here the increase is shown to be statistically significant across all examined tracts on average. Moreover, the earlier observation, based on raw temperature measurements, did not take into account population density or Köppen–Geiger climate zone, variables the ecological regression modeling do account for. Demonstrating that SUHI intensity is increasing through time on average for all population-based groups examined, with a statistically strenuous model, is an important observation and should provide added urgency to mitigation activities directed toward disparities in urban heat exposure.

There is also a statistically significant relationship between the degree of segregation and its effect on SUHI intensity in the inter-city analysis. For every standard deviation increase (0.11) in the *D* index there is on average a corresponding 0.42 K increase in SUHI intensity. The intercept implies that areas with zero degrees of segregation—complete integration—would have a SUHI intensity of 0.59 K and as segregation measures increases so does the heat island temperature intensity. This model similarly contains random effect specifications accounting for influences that are geographically and temporally variable. Observations support Mitchell and Chakraborty where higher measures of the *D* index were shown to indicate increases in urban heat risk for Black and Hispanic communities [6]. Residential segregation tends to compact racial and ethnic groups into densely populated smaller communities, evidenced by the varying number of prevalent census tracts in the study for each group (27,338 White, 6597 Black, 7917 Hispanic, and 2624 Asian prevalent tracts). However, simply integrating these areas within a city, or lowering the level of segregation would not precipitate a lowering of the heat island intensity. There are far too many detrimental environmental impacts of segregation which are difficult to amend and such locations tend to have higher proportions of impervious surface land cover and less tree canopy [55,56]. Simply lowering the level of segregation does not increase tree cover or lower the amount of impervious surface without emphasis placed on urban greening. However, such gentrification initiatives have been shown to continue to relegate historically marginalized groups [57]. Numerous studies have identified “Redlining” or the Home Owners’ Loan Corporation practice beginning in the 1930s of grading neighborhoods for home loans as being a significant driver in the persistence of the observed spatially variable disparities in thermal exposure and components of the physical and social environment that lead to them [56,58,59,60,61]. Significant interventions within the urban environment, that include input from historically marginalized urban populations, will be necessary to lower these observed disparities in SUHI exposure in impactful ways.

### 4.4. Limitations and Caveats

Several limitations of the methodology and approach used in this study should be considered. The primary limitation is the use of 2020 census data to determine the prevalence of each tract from 2003 to 2018. Research has demonstrated the persistence of White–Black, White–Hispanic, and White–Asian segregation in U.S. metropolitan areas through multiple decadal censuses [62,63]. This would imply there is likely not significant enough change in the examined demographic makeup of the census tracts used in this study to significantly affect the results. Potentially, the use of ACS data throughout the timeframe would give a better indication of population prevalence within tracts through time. One would take the ACS releases between 2003 and 2018 and correspond them to the years from the SUHI dataset. However, the number of census tracts or their spatial arrangement for a city can change through time (at each decennial census) and this could potentially add a level of complexity to the models that could affect the comparative ability of the results. This study uses only 2020 data so that SUHI intensity trends in the current tracts identified for each representative prevalent population group could be examined. Therefore, these results show SUHI intensity trends in areas that currently (as of 2020) are prevalent with each population group and not necessarily demonstrative of the comprehensive degree of disparate exposure throughout the timeframe. Future spatial temporal studies might look to incorporate five year ACS data—with consideration of these and other inherent limitations—and compare to results from this analysis.

A further limitation is the spatial resolution of the SUHI dataset, the development of which uses MODIS land surface temperature (MOD11A2, MYD11A2). These SUHI intensity data have a downscaled 300 M resolution and this level likely results in much of the spatial variation at the census tract level being lost, especially in urban areas. Landsat or a finer resolution Earth observation sensor is superior to MODIS for intra-city SUHI intensity studies but lacks the temporal resolution and spatial coverage necessary for a large-scale spatiotemporal study. Utilizing a finer resolution dataset, one could relatively easily incorporate a series of images for a specific city and examine local disparities through time. Results from the current study could be used to identify cities primed for such analysis.

Finally, as noted earlier, the use of prevalence to classify census tracts has certain limitations. Prevalence merely classifies a census tract based on the most populous demographic group within the tract. There are cases where there is a higher population of a secondary group in an alternate primary category than in its neighboring primary grouping. For example, a census tract with a population that is 51% White and 49% Hispanic would be classified as a White prevalent tract. A neighboring tract with 26% Hispanic, 24% White, 25% Black, and 25% Asian would be a Hispanic prevalent tract. Assuming the population of the tract is around 4000 persons the White prevalent tract would have a higher Hispanic population than the designated Hispanic prevalent tract. This is an issue when examining the overall raw trends in SUHI intensity for each group and in the top 25 cities for each group in the dot plots. Prevalence is not used in the models when fitting SUHI intensity with the percentages of each demographic group or the *D* index. Since the modeling, not using the groups, tends to support the trends observed with the groupings, the effect is likely minimal

## 5. Conclusions

This study has contributed to the documentation already present outlining inequitable thermal exposure between different demographic groups [1,2,3,4,5,7]. As evidenced by the SUHI intensity measurements within prevalent census tracts of each demographic group, the temperature disparity is shown to be increasing through time. This increase is statistically significant for both Black and White prevalent census tracts for the observed temperature trend. Overall, within the Bayesian spatial temporal models, temperature is shown to be increasing by 0.01 K per year on average. Granted 0.01 K per year may not seem like an alarming temperature rise but this increase is on average and there are many tracts with greater temperature increases. Further studies should look to examine or grade the tracts that have the highest level of increase or decrease to determine differences between them and help guide intervention strategies aimed at lowering SUHI intensity. Such studies would advance our understanding of social and environmental contributors to thermal exposure at a local scale. Additionally, it would help determine what constitutes a “good” tract vs. a “bad” tract, at least in a thermal sense.

This study has also further demonstrated the degree of difference in heat exposure between the identified population groups. The percentage of the Black population in a census tract is the strongest indicator of increased SUHI intensity of the examined variables. A standard deviation increase in the Black population (23.44%) sees an increase in the average SUHI intensity of a census tract by 0.42 K. Conversely, larger portions of the White population are exposed to lower degrees of SUHI intensity (26.69% increase lowers intensity by 0.25 K). Hispanic and Asian population percentages, similar to Black percentages, align with tendencies toward increasing SUHI intensity. Future studies should look to separate these groups by socioeconomic status (these data were not yet available for the 2020 Census used in this study) to further account for the effect of higher/lower degrees thereof. Finally, this study could be used to identify the most inequitable cities and perform a more local-scale analysis using higher resolution satellite imagery and possibly a finer level of aggregation, such as census block group, for comparison. Such studies could also help determine if many of the trends uncovered in this examination are maintained at a smaller level of aggregation.

Residential segregation was shown to be a statistically significant factor in explaining variability in inter-city SUHI intensity. Higher levels of segregation corresponded to higher SUHI intensity measurements after accounting for climate zone, population density, and spatial and temporal random effects. A 0.11 increase in the *D* index corresponds with an increase in average SUHI intensity by 0.42 K. Future studies could examine other ways of measuring residential segregation or could similarly look at finer-scale segregation in cities such as census tract-level measurements. One could use the block group-level variables identified here to calculate *D* by census tract. Analysis could then determine if this relationship between *D* and SUHI intensity sustains at this scale.

Most important, is combating the inequitable distribution in thermal exposure and its role in environmental injustice. This research underscores potential interactions within the social–physical environment that create extremely hazardous thermal conditions. As global temperatures continue to rise, and as evidenced by the warming quantified in this study, some locations in cities will become “unlivable” during the summer months in the near future [64]. In fact, it may be too late to alter this outcome and the necessity to prepare to mitigate effects of warming is becoming increasingly evident. The primary way for research (such as this) to have the most impact is through affecting policy and therefore it must be brought before policymakers where objective action can be taken, especially for our most vulnerable communities. In this way, research not only elucidates the degrees of injustice and the expectations for the future, but can be used to guide necessary intervention strategies that will make environmental and social inequities less pronounced. To have the most effect, these actions will not only require increased funding, resource allocation in affected communities, and involvement from the scientific community, but will necessitate local participation of the populations that are most inequitably exposed and at the highest risk.

## Figures and Tables

**Table 1:** Descriptive statistics for census tract variables.

Census Tract-Level Descriptive Statistics					
	Percentage White	Percentage Black	Percentage Hispanic	Percentage Asian	SUHI Intensity in K
Minimum	0.00	0.00	0.00	0.00	−13.63
Maximum	100.00	97.64	100.00	100.00	11.06
Mean	51.16	17.87	21.99	8.10	2.05
Standard Deviation	26.69	23.44	23.02	11.60	2.44
	n = 44,476	n = 44,476	n = 44,476	n = 44,476	n = 711,616

**Table 2:** Descriptive statistics of SUHI intensity within group-based prevalent tracts.

SUHI Intensity Descriptive Statistics for Prevalent Tracts of Each Group				
	White	Black	Hispanic	Asian
Minimum	−13.63	−7.64	−7.90	−10.06
Maximum	11.04	11.06	10.26	10.18
Mean	1.46	3.08	3.15	2.73
Standard Deviation	2.29	2.14	2.35	3.12
		(K-S *ρ* = 0.0001)	(K-S *ρ* = 0.0001)	(K-S *ρ* = 0.234)
	n = 410,070	n = 98,955	n = 118,755	n = 39,360

Note: The Kolmogorov–Smirnov (K-S) test statistic was used to compare the White SUHI intensity distribution to the Black, Hispanic, and Asian SUHI intensities. The *ρ* value is the statistical significance of the test statistic determining if the distributions are different.

**Table 3:** Percentage of population (in the study) residing in percentile breakdown of SUHI intensity by census tract.

SUHI Intensity Percentiles					
		White	Black	Hispanic	Asian
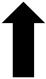	5th	6.32	2.09	3.1	7.17
	10th	13.08	5.37	6.39	11.91
	25th	32.4	17.33	18.33	25.22
Less Than	50th	60.73	41.22	40.9	47.13
Greater Than	75th	15.96	31.7	33.43	29.03
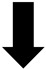	90th	5.43	12.91	14.81	12.72
	95th	2.37	7.06	8.16	6.47
	99th	0.45	1.07	2.24	1.35

**Table 4:** City-level descriptive statistics for each variable.

City-Level Descriptive Statistics						
	Percentage White	Percentage Black	Percentage Hispanic	Percentage Asian	*D*	SUHI Intensity in K
Minimum	0.19	0.01	0.01	0.01	0.08	−2.61
Maximum	90.53	60.40	81.89	49.27	0.63	7.31
Mean	59.16	15.38	16.56	4.75	0.38	1.50
Standard Deviation	14.43	12.74	13.89	5.86	0.11	1.09
	n = 191	n = 191	n = 191	n = 191	n = 191	n = 3056

Note: Black, Asian, and Hispanic prevalent tracts did not exist in all cities, however, each city did contain White prevalent tracts.

**Table 5:** Bayesian hierarchical spatial temporal ecological regression values for posterior β, credibility intervals, Deviance Information Criteria (DIC), and intercepts for each model developed in the study.

	Posterior β for Each Model				
	Independent Variable	Mean β	Credibility Intervals	DIC	Intercept
Model #1	Percentage White	−0.25	(−0.34–−0.17)	-	-
	Percentage Black	0.42	(0.35–0.50)	-	-
	Percentage Hispanic	0.23	(0.17–0.28)	-	-
	Percentage Asian	0.14	(0.10–0.19)	-	-
				766,349,400.40	2.05
Model #2	*D* Multi-Group Dissimilarity Index	0.42	(0.276–0.558)	−11,110.03	0.59

## Data Availability

Data is available through https://dataworks.iupui.edu or upon request.
